# Housing debt and depressive symptoms: evidence from the China family panel studies

**DOI:** 10.1186/s40359-024-01667-z

**Published:** 2024-04-05

**Authors:** Huan Chen, Yuehua Zhang, Kang Cao

**Affiliations:** 1 School of Public Affairs, Zhejiang University, 310058 Hangzhou, China; 2 Innovation Center of Yangtze River Delta, Zhejiang University, 314100 Jiaxing, China; 3 Department of Regional and Urban Planning, Zhejiang University, 310058 Hangzhou, China

**Keywords:** Bank housing loans, Non-bank housing loans, Depressive symptoms, Housing demands

## Abstract

**Background:**

There is limited evidence on the association between housing debt and depressive symptoms in China. This study aimed to examine the impact of housing debt on depressive symptoms and explore the heterogeneous impacts arising from two sources of housing debt and two types of housing demands.

**Methods:**

Using data from the 2016 and 2018 China Family Panel Studies (CFPS), this study included 25,232 Chinese individuals. Depressive symptoms were assessed using the eight-item Center for Epidemiological Studies Depression Scale (CES-D8). Housing debt was measured by dummy variables, indicating whether an individual had housing debt, and continuous variables, which were the logarithm of the total amount of housing debt. The two-way fixed effects model was used to examine the relationship.

**Results:**

Housing debt had a significant positive impact on depressive symptoms in China. Individuals with housing debt had a 0.176-point higher depressive symptom score than those without housing debt. A 10% increase in the total amount of housing debt led to a 0.16-point increase in depressive symptoms. Non-bank housing loans significantly increased the level of depressive symptoms with a larger coefficient (coef = 0.289), while the impact of bank housing loans was small and not statistically significant. In terms of the types of housing demands, a positive impact was observed only among individuals who had only one property meeting their housing consumption demands.

**Conclusions:**

This study found a significant positive impact of housing debt on depressive symptoms, primarily driven by non-bank housing loans. Furthermore, housing debt increased the depressive symptoms among individuals with consumption demands, while those with investment demands did not show a significant impact. Government interventions should prioritize easing formal financial constraints and providing support for individuals with housing consumption demands.

**Supplementary Information:**

The online version contains supplementary material available at 10.1186/s40359-024-01667-z.

## Introduction

In recent years, China has witnessed a significant increase in household debt. From 2007 to 2022, the total amount of household debt increased 14.8-fold from 5.07 trillion yuan to 74.94 trillion yuan. This increase was largely attributed to the rapid escalation of housing prices and demands after the 1998 housing market reform. The total amount of housing loans in China increased remarkably from 0.0485 trillion yuan in 1998 to 38.8 trillion yuan in 2022. Notably, housing loans accounted for 51.8% of household debt in 2022^1^. 2. In addition, informal loans from non-bank institutions, friends, and relatives played a crucial role in Chinese household finance [1, 2]. Over 32% of homeowners purchased houses through informal loans, which had significantly increased housing demand in China [3].

A large number of studies have examined the association between household debt and mental health. Household debt has been found to be associated with anxiety, financial stress, depression, and suicidal ideation [4–12]. However, empirical evidence on the relationship between housing debt and depressive symptoms remains limited. Depressive symptoms refer to a range of emotional, cognitive, and somatic symptoms associated with depression (e.g., feelings of sadness, difficulty sleeping) [13, 14], and serve as effective predictors of clinical depression [15–17]. Only a few studies from the United Kingdom and the United States have focused on housing debt and mental health or depressive symptoms. An earlier study using data from the British Household Panel Survey revealed an association between the onset of mortgage indebtedness and poorer mental health [18]. Among the U.S. homeowners, it was found that individuals with mortgages reported higher levels of psychological distress than those without mortgages [19], and a higher mortgage loan-to-home value ratio led to more depressive symptoms [20]. Similar results have been found in studies focusing on mortgage delinquency and foreclosure [21, 22].

However, there are still gaps in understanding the association between housing debt and depressive symptoms. First, the majority of existing studies have examined the association without addressing endogenous bias. A key factor contributing to endogeneity is self-selection, which arises from omitted variables correlated with the independent variable. This endogeneity can lead to biased estimation results [23, 24]. Relevant studies suggest that there are unobserved individual and household characteristics correlating with household debt and depressive symptoms [11, 20]. In this study, endogeneity concerns arise from the self-selection of the housing debt decision, which may be influenced by unobserved variables correlated with both housing debt and depressive symptoms. For instance, previous studies have shown that credit constraints have a significant impact on homeownership [25–27], household debt [28], and depressive symptoms [29, 30]. These findings suggest that credit constraints may affect both the housing debt decision and the manifestation of depressive symptoms. Second, there is limited evidence on the heterogeneous impacts of housing debt on depressive symptoms. Given the distinct differences between bank and non-bank housing loans, as well as different types of housing demands in China, our study focuses on the heterogeneous impacts arising from two sources of housing debt, namely the bank and non-bank housing loans, and two types of housing demands, namely the consumption and investment demands.

In contrast to the bank housing loans, non-bank housing loans typically feature shorter repayment periods, higher interest rates, and entail ‘human debt’ for borrowers [31, 32]. Only two studies [32, 33] find that non-bank housing loans are associated with less happiness and higher depressive moods, while bank ones are not. However, these studies do not consider endogeneity problems in the estimations. Housing in China has unique nature as it plays a dual role as both a consumption good and an investment tool [34]. This duality leads to two types of housing demands — consumption demands and investment demands — which may result in heterogeneous impacts of housing debt on depressive symptoms. Previous studies have investigated the factors influencing housing demand [35] and their impact on housing prices [36, 37]. However, the heterogeneous impacts of housing debt on depressive symptoms across various housing demands remain to be explored.

Using data from the 2016 and 2018 China Family Panel Studies (CFPS), this study adopted a two-way fixed effects model to examine the impact of housing debt on depressive symptoms. The fixed effects model can reduce endogenous bias by allowing us to control for time-invariant factors, thus resulting in more accurate estimates of the impact. In addition, we distinguished between the sources of housing debt and explored the heterogeneity of bank and non-bank housing loans. Furthermore, we investigated the heterogeneous impacts of housing consumption demands and investment demands based on the number of properties owned by individuals. Finally, a robustness check was conducted. Our study contributes to the existing body of research by mitigating endogeneity bias and emphasizing the significance of considering the sources of housing debt and the types of housing demands.

## Methods

### Data and sample

The data used in this study come from the China Family Panel Studies (CFPS), a nationally representative longitudinal survey of the Chinese population conducted by the Institute of Social Science Survey of Peking University, China [38]. The CFPS aims to collect a wealth of detailed information at the community, family, and individual levels, and to provide comprehensive and objective data on Chinese society. The CFPS has four types of questionnaires - community, family, adult, and child questionnaires - covering a wide range of topics such as community population, family finances, health, education, and marriage. The survey’s primary sampling units (PSUs) were districts in urban areas or counties in rural areas, and a multistage probability proportional to size (PPS) sampling method was used to obtain survey samples across twenty-five provinces in China. In the fieldwork, data were mainly collected by face-to-face interviews aided by computer-assisted personal interviewing (CAPI) technology. The CFPS conducted a baseline survey and interviewed 42,590 individuals from 14,960 households in 2010. Subsequent follow-up surveys were conducted every two years, and six waves of data (collected in 2010, 2012, 2014, 2016, 2018, and 2020) have been published to date. More detailed information and data on the CFPS can be found at the CFPS’s website, http://www.isss.pku.edu.cn/cfps/en.

To ensure consistency in the measurement of depressive symptoms and housing debt^3^, we used CFPS’s data collected in 2016 and 2018, in which 35,975 and 33,973 adults (aged 18 and over) were interviewed, respectively. The study sample was then selected through the following steps. First, we restricted individuals to females aged 18–55 and males aged 18–60, aligning with common age restrictions for housing loans (10,070 in 2016 and 10,404 in 2018 were excluded). Second, individuals with missing information on depressive symptoms, housing debt, or control variables were excluded (6,276 in 2016 and 6,538 in 2018). Third, individuals who did not participate in both the 2016 and 2018 surveys were also excluded (7,013 in 2016 and 4,415 in 2018). In the end, we obtained a balanced panel dataset, comprising a total of 25,232 valid samples, with 12,616 samples in each year.

### Variables

#### Depressive symptoms

The dependent variable in this study is depressive symptoms, measured by the eight-item Center for Epidemiologic Studies Depression scale (CES-D8). The scale was developed by Radloff in 1977 [13] and is one of the most widely used scales to assess depressive symptoms [14, 39]. The CES-D8 consists of five depressive mood items (‘I felt depressed’, ‘I felt that everything I did was an effort’, ‘I felt lonely’, ‘I felt sad’ and ‘I could not get going’), one somatic symptom item (‘my sleep was restless’) and two positive emotion items (‘I was happy’ and ‘I enjoyed life’). For each item, respondents were required to indicate the frequency of various feelings or behaviors in the past week. The response options ranged from ‘rarely or none of the time (less than 1 day)’ to ‘some or a little of the time (1–2 days),’ ‘occasionally or a moderate amount of time (3–4 days),’ and ‘most or all the time (5–7 days).’ In accordance with previous studies [40–42], we first assigned the values of 0–3 to the options for depressive mood and somatic symptom items and the opposite values to the options for positive emotion items. We then calculated the sum of scores for the eight questions, resulting in a range of 0–24, with higher scores indicating more severe depressive symptoms.

#### Housing debt

The independent variable in this study is housing debt. The CFPS questionnaire included detailed information about ‘if an individual has bank loans for housing purchase, construction, or renovation,’ ‘if an individual has loans from other organizations or individuals for housing purchase, construction or renovation,’ and ‘the total amount of outstanding housing debt (principal and interest).’ Based on these questions and following Berger et al. (2016) [11], we measured housing debt in two ways. The first measure was a dummy variable (yes = 1) indicating whether an individual had housing debt. The second measure consisted of the logarithm of the total amount of housing debt. Additionally, recognizing the heterogeneity between bank loans and non-bank loans (loans from friends or other non-bank organizations or individuals, such as private credit institutions, relatives and friends), we also constructed variables to distinguish between these two sources of housing debt.

#### Individual and household characteristics

Following previous studies [33, 42, 43], we included time-varying individual and household characteristics as control variables. Individual characteristics comprised age (in years), education (in years), residence (rural/urban), marital status (married/unmarried), occupation (agricultural work/nonagricultural work), self-rated health status (on a scale of 1 = very bad to 5 = very good), and self-rated socioeconomic status (on a scale of 1 = very low to 5 = very high). Household characteristics included the logarithm of the amount of household income, the logarithm of the amount of household non-housing debt, the number of properties owned, and the number of children.

### Empirical strategy

Using panel data from the CFPS, we adopted the two-way fixed effects model to estimate the impact of housing debt on depressive symptoms. The basic econometric model is specified as follows:

1 $$ {CESD8\_Socre}_{it}={\beta }_{0}+{\beta }_{1}{housing\_debt}_{it}+\gamma {X}_{it}+\delta year+{c}_{i}+{\epsilon }_{it}$$

2 $$\eqalign{& CESD8\_Socr{e_{it}} = {\beta _0} + {\beta _1}Bank\_housing\_loan{s_{it}} \cr & + {\beta _2}Nonbank\_housing\_loan{s_{it}} + \gamma {X_{it}} + \delta year + {c_i} + { \in _{it}} \cr} $$

where $$ {CESD8\_Socre}_{it}$$represents the depressive symptom scores of individual *i* at time *t*; $$ {housing\_debt}_{it}$$ is the independent variable, measured using both dummy variables and continuous variables; $$ {X}_{it}$$ represents various control variables, including individual and household characteristics; 𝑦𝑒𝑎𝑟 represents time fixed effects; $$ {c}_{i}$$ represents individual fixed effects; and $$ {\epsilon }_{it}$$ is the random error.

As shown in Eq. (2), we also conducted regressions distinguishing the two sources of housing debt to explore their heterogeneous impacts. Compared to standard ordinary least squares (OLS) regressions, the fixed effects model enabled us to control for time-invariant omitted variables, thereby enhancing the understanding of the causal relationship between housing debt and depressive symptoms.

## Results

### Descriptive statistics

Table 1 presents the descriptive statistics of the study participants. The results show that the mean CES-D8 scores in 2016 and 2018 were 4.966 and 5.464, respectively. In terms of housing debt, the percentage of participants with housing debt increased from 24.1% in 2016 to 25% in 2018. Furthermore, the mean value of the amount of housing debt increased from 34,905 yuan to 50,179 yuan, indicating a continuous growth of housing debt in China. In 2016, participants were on average 39 years old with middle school education (nine years). Approximately half of the participants lived in urban areas (49.4% in 2016 and 51.9% in 2018). The majority of participants were married (82.2% in 2016 and 83.4% in 2018). About one-third of the participants engaged in agricultural work (35.3% in 2016 and 34.1% in 2018). The mean values of self-rated health status and socioeconomic status were about three. Regarding household characteristics, the average household income increased from 82,686 yuan in 2016 to 102,504 yuan in 2018, and the average household non-housing debt increased from 14,849 yuan in 2016 to 19,115 yuan in 2018. In 2018, participants owned an average of 1.151 properties and had an average of 1.360 children.

**Table 1:** Descriptive statistics of the study participants

Variables	2016			2018	
	Mean	S.D.		Mean	S.D.
Dependent variable					
Depressive symptoms (CES-D8 scores)	4.966	3.747		5.464	3.809
**Independent variable**					
Housing debt (Yes = 1)	0.241	0.428		0.250	0.433
Bank housing loans (Yes = 1)	0.118	0.322		0.144	0.351
Non-bank housing loans (Yes = 1)	0.167	0.373		0.159	0.366
The amount of housing debt (yuan)	34,905	136,560		50,179	175,411
The amount of bank housing loans (yuan)	24,063	124,639		39,637	165,131
The amount of non-bank housing loans (yuan)	12,419	183,062		10,541	44,109
**Control variables**					
Age	39.13	11.02		41.01	11.09
Education	8.909	4.499		9.137	4.623
Residence (Urban = 1)	0.494	0.500		0.519	0.500
Marital status (Married = 1)	0.822	0.382		0.834	0.372
Occupation (Agricultural work = 1)	0.353	0.478		0.341	0.474
Health status	3.113	1.171		3.074	1.168
Socioeconomic status	2.743	1.005		3.026	1.009
The total household income (yuan)	82,686	179,808		102,504	146,180
The total household non-housing debt (yuan)	14,849	75,156		19,115	102,386
The number of properties owned	1.129	0.600		1.151	0.660
The number of children	1.332	0.950		1.360	0.931
**Observations**	12,616			12,616	

*Note* In the regressions, we used the logarithm of the amount of debt and income

Table 2 reports the difference in CES-D8 depressive symptom scores between samples with and without housing debt using T-tests. In both 2016 and 2018, the mean depressive symptom scores for samples with housing debt were higher than those without debt, and the differences between the two groups were statistically significant at the 1% level. When data from both years were combined, the mean depressive symptom score for samples with housing debt was significantly higher by 0.410 points compared to samples without housing debt. This result suggested that housing debt was positively associated with higher depressive symptoms. However, further regression analysis is needed to examine the causal relationship.

**Table 2:** Descriptive statistics of depressive symptoms

Variables	With housing debt			Without housing debt			T-test	
	(1)	(2)		(3)	(4)		(5)	(6)
	Obs.	Mean		Obs.	Mean		Difference	S.D.
CES-D8 scores in 2016	3038	5.240		9578	4.879		0.361***	0.078
CES-D8 scores in 2018	3153	5.796		9463	5.353		0.443***	0.078
CES-D8 scores in 2016 and 2018	6191	5.524		19,041	5.114		0.410***	0.055

*Note>* *** *p* < 0.01

### The impact of housing debt on depressive symptoms

Table 3 presents the results from two-way fixed effects models on the impact of housing debt on depressive symptoms. The findings in Column (1) indicate that individuals with housing debt had a 0.176-point higher depressive symptom score than those without housing debt. Column (3) shows that for every 10% increase in the total amount of housing debt, the depressive symptom score increased by 0.16 points. These effects were significant at the 5% level. Therefore, after controlling for time-invariant omitted variables and time-varying observed variables, we found a significant positive impact of housing debt on depressive symptoms.

**Table 3:** The impact of housing debt on depressive symptoms (CES-D8 scores)

Variables	(1)	(2)	(3)	(4)
**Housing debt (Yes = 1)**	0.176**			
	(0.072)			
Bank housing loans (Yes = 1)		0.053		
		(0.106)		
Non-bank housing loans (Yes = 1)		0.213***		
		(0.079)		
**Ln (amount of housing debt)**			0.016**	
			(0.007)	
Ln (amount of bank housing loans)				0.005
				(0.009)
Ln (amount of non-bank housing loans)				0.019**
				(0.007)
Control variables	Yes	Yes	Yes	Yes
Individual fixed effects	Yes	Yes	Yes	Yes
Time fixed effects	Yes	Yes	Yes	Yes
Observations	25,232	25,232	25,232	25,232

*Note* Robust standard errors are in parentheses. *** *p* < 0.01, **p < 0.05, * *p* < 0.1. The complete table is in the Appendix Table A1

### Heterogeneity analysis of bank and non-bank housing loans

To examine the heterogeneous impacts of the sources of housing debt, we conducted regressions distinguishing bank and non-bank loans. The results in Columns (2) and (4) of Table 3 indicate that the impact of housing debt on depressive symptoms was predominantly driven by non-bank housing loans. The impact of bank housing loans was small and not statistically significant, while non-bank housing loans significantly increased depressive symptoms with a larger coefficient. Specifically, Column (2) shows that individuals with non-bank housing loans exhibited a 0.213-point increase in depressive symptoms. Furthermore, a 10% increase in non-bank housing loans increased depressive symptoms by 0.19 points. The heterogeneous impacts can be attributed to the large differences in repayment methods, interest rates, and the concept of ‘human debt’ between bank and non-bank loans.

First, in terms of repayment methods, bank housing loans generally used real estate as collateral, and borrowers made regular repayments of principal and interest to the bank. Bank housing loans have longer repayment periods than other types of loans, and the installment payments can help distribute financial stress. In contrast, the repayment periods of the non-bank loans are relatively shorter, concentrating the psychological pressure on borrowers over a short period of time. Previous research had revealed that the positive relationship between housing debt and higher depressive symptoms was mainly driven by short-term debt, while medium- and long-term debt were not significantly associated with depressive symptoms [11]. Second, the interest rates on loans from private credit institutions were much higher than those from banks in China [31]. Third, individuals who borrowed money from relatives and friends incurred what is known as ‘human debt’ [32] or ‘debt of gratitude’ [33], which may put more psychological pressure on them. As a result, non-bank housing loans were strongly associated with higher depressive symptoms compared to bank housing loans.

## Discussion

### Heterogeneity analysis of housing consumption and investment demands

Individuals build or purchase properties to satisfy consumption or investment demands, and these two different housing purposes may lead to heterogeneous impacts of housing debt on depressive symptoms. We hypothesized that individuals with only one property were more likely to meet consumption demands. Therefore, housing debt incurred by these individuals would induce more psychological pressure on them and significantly increase their depressive symptoms. In contrast, those with two or more properties were more likely to view their properties as investment tools, thus resulting in a diminished impact of housing debt on their depressive symptoms. To test this hypothesis, we divided our sample into two subsamples based on the number of properties owned and subsequently examined the heterogeneous impacts.

In Table 4, Columns (1)-(4) present results for the sample with only one property, while Columns (6)-(8) present results for the sample with two or more properties. Among individuals with consumption demands, housing debt was associated with a 0.289-point increase in depressive symptoms, and a 10% increase in the amount of housing debt resulted in a 0.28-point increase in depressive symptoms. In addition, the positive impact was primarily observed with non-bank housing loans rather than bank housing loans, which is similar to the results in Table 3 but with larger estimated coefficients. In terms of investment demands, housing debt was not associated with depressive symptoms among individuals who owned more than two properties. These findings support our hypothesis that the impacts of housing debt on depressive symptoms were heterogeneous according to housing consumption and investment demands.

**Table 4:** Heterogeneous impact of housing consumption and investment demand

Variables	Sample with only one property (Consumption demand)					Sample with two or more properties (Investment demand)			
	(1)	(2)	(3)	(4)		(5)	(6)	(7)	(8)
**Housing debt (Yes = 1)**	0.289***					-0.158			
	(0.098)					(0.221)			
Bank housing loans (Yes = 1)		-0.012					-0.066		
		(0.160)					(0.273)		
Non-bank housing loans (Yes = 1)		0.396***					-0.047		
		(0.104)					(0.228)		
**Ln (amount of housing debt)**			0.028***					-0.013	
			(0.009)					(0.019)	
Ln (amount of bank housing loans)				-0.0003					-0.005
				(0.014)					(0.023)
Ln (amount of non-bank housing loans)				0.038***					-0.009
				(0.010)					(0.021)
Control variables	Yes	Yes	Yes	Yes		Yes	Yes	Yes	Yes
Individual fixed effects	Yes	Yes	Yes	Yes		Yes	Yes	Yes	Yes
Time fixed effects	Yes	Yes	Yes	Yes		Yes	Yes	Yes	Yes
Observations	18,143	18,143	18,143	18,143		4827	4827	4827	4827

*Note* Robust standard errors are in parentheses. *** *p* < 0.01

### Robustness check

In the previous empirical analysis, we measured depressive symptoms using the CES-D8 score, which ranges from 0 to 24. To mitigate potential measurement error, we conducted a robustness check using a validated cut-off of 9, following previous studies [44–46]. The results are presented in Table 5. Panel A shows a significant positive impact of housing debt on depressive symptoms. This impact was mainly driven by non-bank housing loans, while the association between bank housing loans and depressive symptoms was not statistically significant. Panel B and Panel C present the results using the sample with only one property and two or more properties, respectively. Consistent with the results in Table 4, Panel B reveals that housing debt, especially non-bank housing loans, was associated with higher depressive symptoms among individuals with only one property for consumption demands. Conversely, in Panel C, we found no significant association between housing debt and depressive symptoms among individuals with two or more properties for investment demands. These results are in align with the baseline estimates, suggesting that our findings are robust.

**Table 5:** Robustness check: a validated cut-off of 9 for CES-D8 scores

Variables	(1)	(2)	(3)	(4)
Panel A: Full sample				
**Housing debt (Yes = 1)**	0.017**			
	(0.008)			
Bank housing loans (Yes = 1)		0.012		
		(0.011)		
Non-bank housing loans (Yes = 1)		0.018**		
		(0.008)		
**Ln (amount of housing debt)**			0.002**	
			(0.001)	
Ln (amount of bank housing loans)				0.001
				(0.001)
Ln (amount of non-bank housing loans)				0.002**
				(0.001)
Observations	25,232	25,232	25,232	25,232
***Panel B: Sample with only one property (consumption demand)***				
**Housing debt (Yes = 1)**	0.025**			
	(0.010)			
Bank housing loans (Yes = 1)		-0.0005		
		(0.017)		
Non-bank housing loans (Yes = 1)		0.034***		
		(0.011)		
**Ln (amount of housing debt)**			0.003***	
			(0.001)	
Ln (amount of bank housing loans)				0.0001
				(0.001)
Ln (amount of non-bank housing loans)				0.003***
				(0.001)
Observations	18,143	18,143	18,143	18,143
***Panel C: Sample with two or more properties (investment demand)***				
**Housing debt (Yes = 1)**	-0.003			
	(0.024)			
Bank housing loans (Yes = 1)		0.039		
		(0.026)		
Non-bank housing loans (Yes = 1)		-0.028		
		(0.027)		
**Ln (amount of housing debt)**			-0.0001	
			(0.002)	
Ln (amount of bank housing loans)				0.003
				(0.002)
Ln (amount of non-bank housing loans)				-0.003
				(0.002)
Observations	4827	4827	4827	4827

*Note* Control variables, individual and time fixed effects were included in regressions. Robust standard errors are in parentheses. *** *p* < 0.01, **p < 0.05, * *p* < 0.1

### Limitations

This study has several limitations. First, despite employing panel data and fixed effects models, endogeneity concerns still exist in identifying the causal relationship between debt and depressive symptoms. Although fixed effects models can control for time-invariant omitted variables and time-varying observed variables, our estimation results may be biased due to time-varying omitted variables. Second, previous studies have shown that in addition to the amount of debt, subjective financial stress, well-being, and worry play an important role in the association between debt and depression [5, 47, 48]. Unfortunately, the CFPS did not collect information on subjective financial stress. Therefore, we were unable to examine this association. Third, we focused on depressive symptoms in the general population and therefore used the CES-D as our measure. However, due to data limitations, we did not include clinical depression in our analysis. Although depressive symptoms are effective predictors of clinical depression [15–17], it should be noted that they are not synonymous.

## Conclusion

This study found that housing debt had a significant positive impact on depressive symptoms. We distinguished the sources of housing debt and found that non-bank housing loans were associated with increased depressive symptoms, whereas bank housing loans did not exhibit such an association. Furthermore, our results revealed heterogeneity based on housing consumption and investment demands. While housing debt significantly increased depressive symptoms for individuals with consumption demands, it did not affect those with investment demands.

The rapid growth of housing debt and the risk of depression in China has attracted much attention from researchers to policymakers. This study contributes to a better understanding of the association between housing debt and depressive symptoms and its heterogeneity. Accordingly, our findings provide empirical evidence to support policies concerning the housing market in three ways. First, the state of the housing market is closely linked to the risk of depression in the general population. Policies should be released and implemented to promote the healthy development of the housing market, thereby reducing housing debt and alleviating depressive symptoms. Second, the positive impact on depressive symptoms is primarily attributed to non-bank housing loans rather than bank housing loans. Therefore, improving accessibility to formal credit can contribute to mitigating this impact. Third, better targeting of housing support policies can help reduce mental health inequalities. As individuals with housing consumption demands suffer from more severe depressive symptoms due to housing debt, future policies should prioritize these individuals.

### Electronic supplementary material

Below is the link to the electronic supplementary material.

Supplementary Material 1

## Data Availability

The datasets analyzed during this study are available at http://www.isss.pku.edu.cn/cfps/en/.

## Footnotes

1 Data on housing loans in 1998 come from a report by the Asian Development Bank and are available at https://www.adb.org/sites/default/files/project-documents//moc-hou-cn.pdf. Other data come from the *Statistical Report on the Credit Structure of Financial Institutions* of the People’s Bank of China.

2 Depression symptoms measured in the 2010 and 2014 questionnaires differ from those measured in the 2016 and 2018 questionnaires, and housing debt measured in the 2010 and 2012 questionnaires differs from those measured in the 2016 and 2018 questionnaires. In response to the COVID-19 pandemic, the CFPS prioritized telephone interviews in 2020, resulting in declined response rates and potentially inaccurate responses. In addition, the impact of COVID-19 may lead to increased estimation bias in fixed effects models. Therefore, we did not use the data collected in 2020.

3 Depression symptoms measured in the 2010 and 2014 questionnaires differ from those measured in the 2016 and 2018 questionnaries, and housing debt measured in the 2010 and 2012 questionnaries differs from those measured in the 2016 and 2018 questionnaries. In response to the COVID-19 pandemic, the CFPS prioritized telophone interviews in 2020, resulting in declined response rates and potentially in accurate responses. In addition, the impact of COVID-19 may lead to increased estimation bias in fixed effects models. Therefore, we did not use the data collected in 2020.
