# Correction: Housing debt and depressive symptoms: evidence from the China family panel studies

**DOI:** 10.1186/s40359-024-01757-y

**Published:** 2024-05-14

**Authors:** Huan Chen, Yuehua Zhang, Kang Cao

**Affiliations:** 1 School of Public Affairs, Zhejiang University, Hangzhou, 310058 China; 2 Innovation Center of Yangtze River Delta, Zhejiang University, Jiaxing, 314100 China; 3 Department of Regional and Urban Planning, Zhejiang University, Hangzhou, 310058 China

**Correction: BMC Psychology (2024) 12:186**

10.1186/s40359-024-01667-z

Following publication of the original article, the authors identified three typographical errors. First, in the first paragraph of the ‘Data and Sample’ section: ‘The CFPS conducted a baseline survey and interviewed 42,590 individuals from 14,960 households in 2020’, where ‘2020’ needed to be corrected to ‘2010’. Second, in the explanation of the variable ‘Housing debt’: ‘if an individual has loans from other organizations or individuals for housing purposes, construction or renovation’, where ‘purposes’ needed to be corrected to ‘purchase’. Third, the columns of Table 2 were number 3 to 8, whereas they should be numbered 1 to 6.

The published article has been updated to amend these errors. The authors thank you for reading this erratum and apologize for any inconvenience caused.

