# Supplementary material for: Housing debt and depressive symptoms: evidence from the China family panel studies
Source: BMC Psychol. 2024 Apr 5;12:186. doi: 10.1186/s40359-024-01667-z (PMC10996272; doi:10.1186/s40359-024-01667-z)
Supplement: Supplementary file 1 — Supplementary Material 1 [file 40359_2024_1667_MOESM1_ESM.docx]

**Appendix**

**Table A1 The impact of housing debt on depressive symptoms (CES-D8 scores)**

| **Variables** | **(1)** | **(2)** | **(3)** | **(4)** |
| --- | --- | --- | --- | --- |
| **Housing debt (Yes=1)** | 0.176** |  |  |  |
|  | (0.072) |  |  |  |
| Bank housing loans (Yes=1) |  | 0.053 |  |  |
|  |  | (0.106) |  |  |
| Non-bank housing loans (Yes=1) |  | 0.213*** |  |  |
|  |  | (0.079) |  |  |
| **Ln (amount of housing debt)** |  |  | 0.016** |  |
|  |  |  | (0.007) |  |
| Ln (amount of bank housing loans) |  |  |  | 0.005 |
|  |  |  |  | (0.009) |
| Ln (amount of non-bank housing loans) |  |  |  | 0.019** |
|  |  |  |  | (0.007) |
| Age | 0.272*** | 0.271*** | 0.272*** | 0.271*** |
|  | (0.099) | (0.099) | (0.099) | (0.099) |
| Education | -0.004 | -0.004 | -0.004 | -0.004 |
|  | (0.035) | (0.035) | (0.035) | (0.035) |
| Residence (Urban=1) | 0.308** | 0.312** | 0.308** | 0.317** |
|  | (0.154) | (0.154) | (0.154) | (0.154) |
| Marital status (Married=1) | -0.830*** | -0.829*** | -0.832*** | -0.831*** |
|  | (0.205) | (0.205) | (0.205) | (0.205) |
| Occupation (Agricultural work=1) | 0.315*** | 0.317*** | 0.316*** | 0.317*** |
|  | (0.114) | (0.114) | (0.114) | (0.114) |
| Health status | -0.400*** | -0.400*** | -0.400*** | -0.401*** |
|  | (0.031) | (0.031) | (0.031) | (0.031) |
| Socioeconomic status | -0.171*** | -0.171*** | -0.171*** | -0.171*** |
|  | (0.032) | (0.032) | (0.032) | (0.032) |
| The total household income (yuan) | 0.018 | 0.018 | 0.017 | 0.018 |
|  | (0.043) | (0.043) | (0.043) | (0.043) |
| The total household non-housing debt (yuan) | 0.017** | 0.017** | 0.017** | 0.017** |
|  | (0.007) | (0.007) | (0.007) | (0.007) |
| The number of properties owned | -0.138*** | -0.136*** | -0.141*** | -0.136*** |
|  | (0.051) | (0.052) | (0.051) | (0.052) |
| The number of children | 0.151* | 0.152* | 0.151* | 0.153* |
|  | (0.085) | (0.085) | (0.085) | (0.085) |
| Individual fixed effects | Yes | Yes | Yes | Yes |
| Time fixed effects | Yes | Yes | Yes | Yes |
| Observations | 25232 | 25232 | 25232 | 25232 |

*Note*: Robust standard errors are in parentheses. *** p<0.01, **p<0.05, * p<0.1.
